# Effect of temperature-sensitive nanogel combined with angioplasty on sICAM-1 and VE-cadherin in lower extremity arterial occlusion rabbits

**DOI:** 10.1080/21655979.2021.2009967

**Published:** 2021-12-21

**Authors:** Ming Qi, Ze Song, Lei Wang, Xu Xie

**Affiliations:** Department of Vascular Surgery, The First Affiliated Hospital of Dalian Medical University, Dalian Liaoning Province

**Keywords:** Vascular embolization agent, temperature-sensitive nanogel, subintimal angioplasty, lower extremity arterial occlusion

## Abstract

The study was to explore the effect of subintimal angioplasty (SIA) on the levels of soluble intercellular adhesion molecule-1 (sICAM-1) and vascular endothelial cadherin (VE-cadherin) in the rabbit model of lower extremity arterial occlusion. Specifically, the poly(N-isopropylacrylamide-co-butyl methacrylate) (PIB) temperature-sensitive nanogel was prepared, and the cytotoxicity of direct and indirect contact with PIB temperature-sensitive gel was analyzed then. Subsequently, the PIB temperature-sensitive gel was injected to the New Zealand white rabbit to prepare the lower extremity arterial occlusion model. The healthy control, model group, and SIA group were compared for the serum lipids, fibrinogen (Fbg), fibrinogen (Fbg), and fibrinogen (Fbg) levels. The results showed that the cell proliferation activity and survival rate were always higher than 90% under different concentrations of PIB temperature-sensitive gels. Compared with the model group, the SIA group had increased total cholesterol (TC), triglycerides (TG), low-density lipoprotein (LDL), and Fbg levels, but decreased high-density lipoprotein (HDL) level (*P < *0.05); decreased TXB2, ET-1, and ICAM-1 levels, but increased levels of 6-Keto-PGF1α and NO (*P < *0.05); and decreased sICAM-1 and VE-cadherin levels (*P < *0.05). It showed that PIB temperature-sensitive nanogel can elicit vascular embolism, and SIA is suggested in the treatment of lower extremity arterial occlusion.

## 1. Introduction

Atherosclerosis occlusion is a common vascular disease mainly caused by long-term chronic ischemia, thus resulting in the insufficient blood supply to the arteries of the distal limbs of the patient. Once developing into the advanced stage, it will seriously affect the patient’s quality of life [1,2]. Its incidence is increasing year by year, so it is necessary to explore effective treatment methods for this disease.

The atherosclerosis occlusion is associated with the endothelial function injury, thrombus formation, vascular remodeling, and endothelial cell migration, resulting in local inflammation and the release of cytokines [3,4]. Vascular endothelial cadherin (VE-cadherin) is an important indicator to evaluate vascular endothelial function [5]. Intercellular adhesion molecules-1 (ICAM-1) is up-regulated during vascular injury and inflammation, and is closely related to vascular endothelial injury [6].

Interventional embolization therapy belongs to the category of interventional radiology, and the choice of vascular embolization agents directly determines the therapeutic effects [7]. N-isopropylacrylamide is a temperature-sensitive vascular embolization material, and it has achieved good results in vascular interventional therapy [8]. Nevertheless, it can also produce toxic substances through hydrolysis. As nano-technology marches forward continuously, nano-materials have been widely used in various fields [9,10].

Nanomaterials have the characteristics of non-toxicity, local tissue reaction, and long maintenance time, which can solve the above problems. Ideal vascular embolization agents are made from nanomaterials. However, few studies have used nanomaterials to prepare vascular embolization agents, and to evaluate their clinical application value. In this study, a temperature-sensitive nanogel was prepared and its cytotoxicity was evaluated. Subsequently, it was used as a vascular embolization agent to prepare an animal model of lower extremity arterial occlusion. Finally, the changes in serum lipids, fibrinogen, and endothelial function indexes were evaluated before and after subintimal angioplasty (SIA). The results of this study were expected to provide a theoretical basis for the development of new temperature-sensitive nanogels used as vascular embolization agents, so as to improve the clinical efficacy of upper and lower extremity arterial occlusion.

## 2. Materials and methods

### 2.1 Experimental materials

DMEM medium, fetal bovine serum, and penicillin streptomycin mixture were purchased from Gibico, USA; PIB temperature-sensitive nanogel preparation reagents (analytical purity) were purchased from Tianjin Komiou Chemical Reagent Development Center; CCK-8 reagent kit was purchased from Abcam, China; Calcein-AM and PI staining solutions were purchased from Sigma, USA; and ELISA kit were purchased from Sigma, USA.

### 2.2 Synthesis of PIB temperature-sensitive nanogel

In this study, the preparation method refers to precipitation polymerization, and it is slightly modified. 2.263 g of 20 mmol N-isopropylacrylamide (NIPAM) and 0.168 mL 1 mmol butyl methacrylate (BMA) were dissolved in 170 mL ultrapure water, together with 0.0324 g of 0.2 mmol N,N’-methylenebisacrylamide (MBA) and 0.032 g of 0.65 mmmol sodium dodecyl sulfate (SDS). The mixture was then transferred to a three-neck round bottom flask equipped with reflux condenser and air duct. It was stirred at 40°C until fully dissolved, followed by injecting nitrogen for 1 h. At 70°C, 0.095 g of 2 mmol potassium persulfate (KPS) was added for polymerization and after 4.5 h, 2.0 g-grade poly(N-isopropylacrylamide-co-butyl methacrylate) (PIB) dispersion was obtained. The dispersion was then dialyzed to obtain the final PIB temperature-sensitive nano-gel.

### 2.3 Characterization of PIB

The ultrapure water was used to dilute iohexol at a ratio of 1:1, and then, PIB nanogel was added overnight to fully swell it, so as to obtain the PIB nanogel-iohexol dispersion containing 6% (wt/wt) PIB. The prepared embolic agent was then transferred into a glass bottle for a water bath (25°C ~ 40°C), and the phase transition temperature was measured using the visual method and the bottle inversion method.

A 2.7 F microcatheter was used to inject the PIB nanogel, and the phase transition process was observed, to record the time it takes to completely transform from the sol state to the gel.

### 2.4 Cytotoxicity of indirect contact with PIB thermosensitive nanogel

0.5 mL of the 6% (wt/wt) PIB nanogel iohexol dispersion was transferred into a 24-well plate, followed by incubation at 37°C until the phase of PIB changed. 1.6 mL DMEM medium containing 10% fetal bovine serum and 1% penicillin-streptomycin was incubated for 24 hours. Then, the supernatant was collected to harvest the 100% extract, which was diluted to 25% and 50% with DMEM medium. The third-generation Hela cells were inoculated in a 96-well plate. When the cell confluence reached about 80%, PIB extracts of different concentrations were added. Subsequently, a blank control group (only DMEM medium, no cells) and a negative control group (cells + DMEM medium) were set and incubated for 3 days. Reagents were added per the CCK-8 kit instructions, and a multi-function microplate reader (BIO-TEK, USA) was used to detect the absorbance of cells in each well. The relative cell proliferation rate was calculated as follows: (OD value of the treatment group-OD value of the blank control group)/(OD value of the negative control group-OD value of the blank control group) × 100%.

### 2.5 Cytotoxicity of direct contact with PIB thermosensitive nanogel

The PIB freeze-dried powder was dissolved in DMEM medium to obtain PIB nanogel dispersions with concentrations of 10 mg/mL and 20 mg/mL. The coverslip was coated with 0.1 mg/mL poly-lysine. Hela cells were then transferred in a 24-well plate. After cell adherence, PIB-containing DMEM medium was added. At the same time, a negative control group (cells+normal DMEM medium) and a blank control (only DMEM medium) were set and incubated at 37°C for 48 hours. After washed with PBS, the cells were immersed in 5 μg/mL Calcein-AM and 6 μg/mL PI, followed by incubation at 37°C in the dark for 20 min. Next, the supernatant was discarded, and the cells were washed with PBS 3 times. Finally, 5 fields of view were selected and visualized under a laser confocal microscope (Olympus Japan). Specific operation steps follow the kit instructions. The cell survival rate was calculated as follows: number of living cells/total number of cells×100%.

### 2.6 Animal models of lower extremity arteriosclerosis occlusion

New Zealand white rabbits were used, with no limitations to the gender. They were intraperitoneally injected with 30 mg/kg 3% sodium pentobarbital solution for anesthesia. Next, the completely anesthetized rabbits were fixed on the operating table in a supine position, with the lateral groin and hind limb skin disinfected with iodophor. A 1 ~ 2 cm longitudinal incision was made in the groins on both sides to separate the femoral artery. A 2.7 F microcatheter was used to inject PIB nanogel at a rate of 0.1 mL/s to embolize the lower extremity arteries. Then, the incision was sutured with No. 0 thread. Next, 10 mL saline was subcutaneously injected into the back. The rabbits were put back in the cage after the vital signs returning to normal. They fed high-fat feed (50 kg basic feed + 1 kg egg yolk powder + 5 kg lard + 750 g cholesterol + 100 g sodium cholate + 1 g methylthiouracil), and were injected with 3 × 10^5^ U/kg VD3 intramuscularly.

According to the surgical methods, the rabbits were randomly divided into:

(1) Control group (n = 12): healthy rabbits without any treatment and routinely reared.

(2) Model group (n = 12): rabbits of lower extremity arteriosclerosis occlusion and fed with high-fat diet.

(3) SIA group (n = 12): rabbits with lower extremity arteriosclerosis occlusion, intraperitoneally injected with 5 mL/kg 5% chloral hydrate solution for anesthesia, followed by sub-membrane Angioplasty (SIA) treatment.

### 2.7 Observation indicators

With healthy rabbits as the control group, blood from the ear veins was collected from 6 experimental rabbits at 6 weeks and 12 weeks after the operation. The automatic biochemical analyzer was used to detect serum total cholesterol (TC), triglycerides (TG), high-density lipoprotein (HDL), low-density lipoprotein (LDL), and fibrinogen (Fbg), followed by double-antibody two-step sandwich enzyme-linked immunosorbent assay to detect TXB2, 6-Keto-PGF1α, ET-1, NO, ICAM-1, VE-cadherin, and sICAM-1.

### 2.8 Statistics

All experimental data were expressed as mean ± standard deviation ($\overset{ˉ}{x}\pm s$), and SPSS 22.0 was used to process experimental results. Differences between groups were compared using independent sample *t* test, *F*-test, and one-way analysis of variance. A value of *P < *0.05 was the threshold for significance.

## 3. Results

In this study, PIB temperature-sensitive nanogel was successfully prepared by precipitation polymerization, and the cytotoxicity of PIB nano-gel was evaluated by CCK-8 and Calcein-AM/PI staining. Then, it was used as a vascular embolization agent to prepare animal models of lower extremity arterial occlusion, and the changes of serum lipid, fibrinogen, and endothelial function indexes were evaluated before and after SIA.

### 3.1 Characterization of PIB temperature-sensitive nanogel

**Figure 1.**
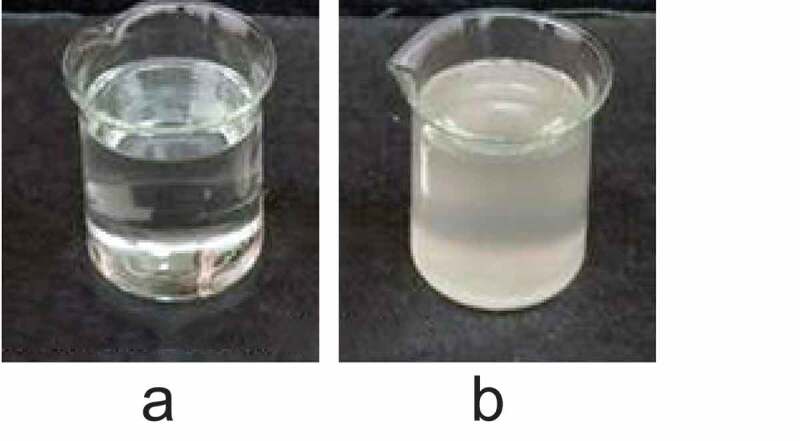
Characterization of PIB temperature-sensitive nanogel

The PIB temperature-sensitive nanogel was characterized, as shown in Figure 1. It was noted from Figure 1(a) that the PIB temperature-sensitive nanogel was a transparent liquid sol at room temperature and had good fluidity. It was noted from Figure 1(b) that, when the temperature exceeded 36°C, the PIB temperature-sensitive nanogel transformed into a white solid gel.

### 3.2 Cytotoxicity analysis of PIB thermosensitive nanogel

**Figure 2.**
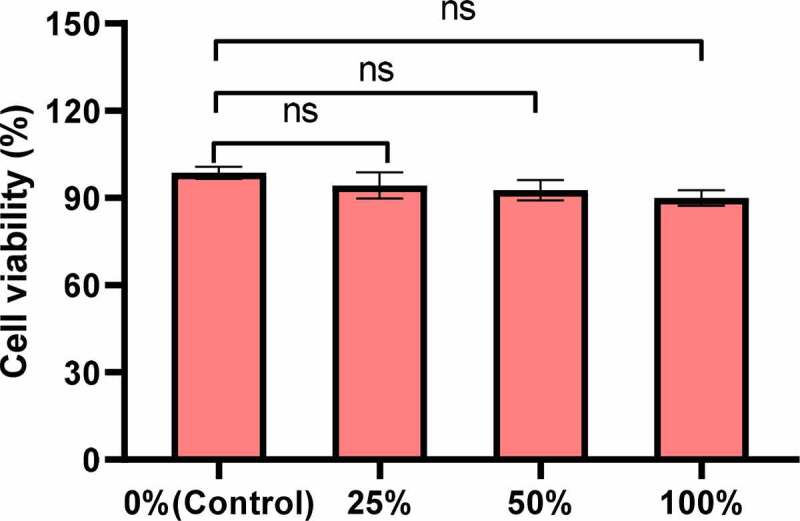
The proliferation activity effect under different concentrations of PIB temperature-sensitive nanogel extracts. (^ns^*P>*0.05)

First, the effect of PIB temperature-sensitive nanogel extracts of different concentrations on the proliferation activity of Hela cells was analyzed. The CCK-8 test results showed that, compared with the control group, the cell proliferation activity showed a downward trend under 25%, 50%, and 100% PIB temperature-sensitive nanogel extracts, and the decline was more obviously under the 100% concentration. However, the proliferation activity was always higher than 90%, and after comparison, and no notable differences were noted between groups (*P > *0.05) (Figure 2).

**Figure 3.**
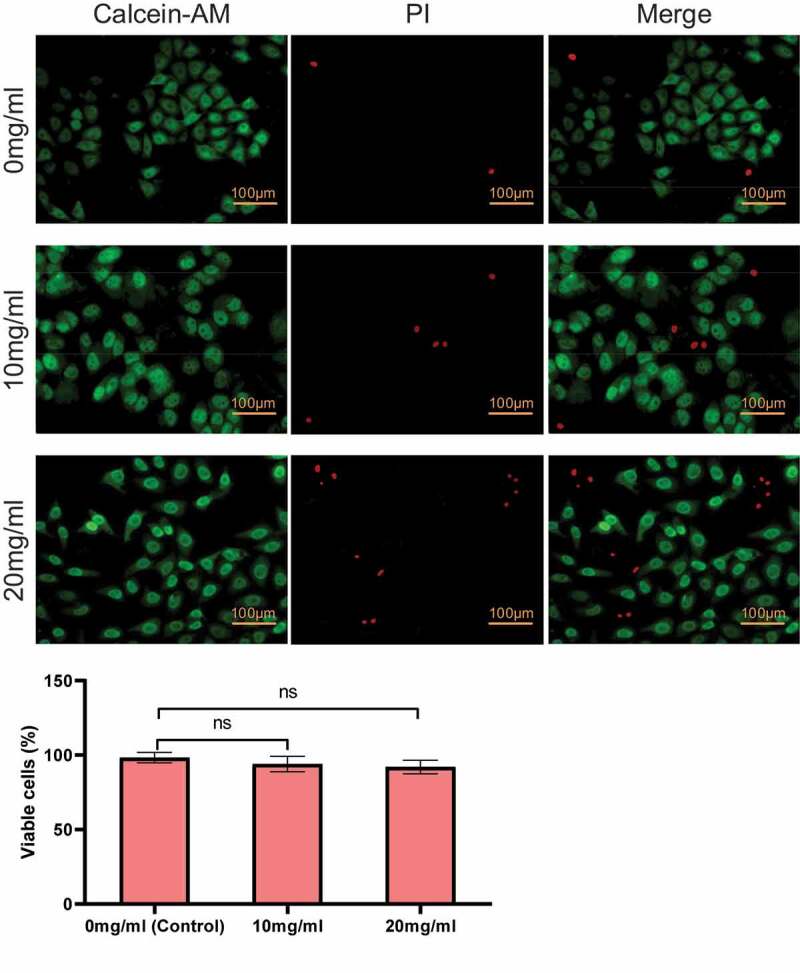
Cell apoptosis under different concentrations of PIB temperature-sensitive nanogels. (^ns^*P>*0.05)

Secondly, the cytotoxicity of direct contact with PIB thermosensitive nanogel was analyzed. As shown in Figure 3, the increase of the concentration of PIB led to a lifted number of cells stained red. However, compared with 0 mg/mL (control group), there was no notable difference between 10 mg/mL and 20 mg/mL in the apoptosis rate (*P > *0.05).

### 3.3 Determination of blood lipids and fibrin

**Figure 4.**
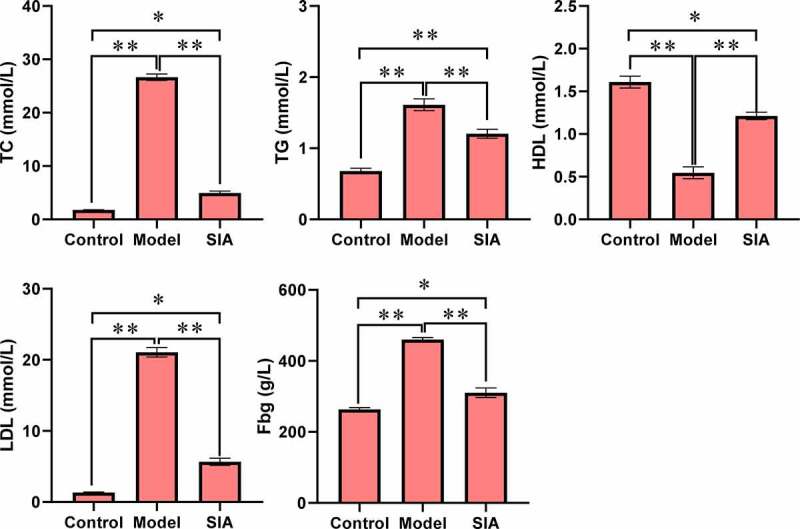
The serum lipids and fibrinogen levels. (*P < 0.05; **P < 0.01)

As shown in Figure 4, compared with the control group, the levels of TC, TGLDL, and Fbg in the model group and the SIA group increased, while the HDL level decreased, and the difference was notable (*P < *0.05). Compared with the model group, serum TC, TGLDL, and Fbg levels in the SIA group decreased, while the HDL level increased, and the difference was notable (*P < *0.05).

### 3.4 Measurement of other indicators

**Figure 5.**
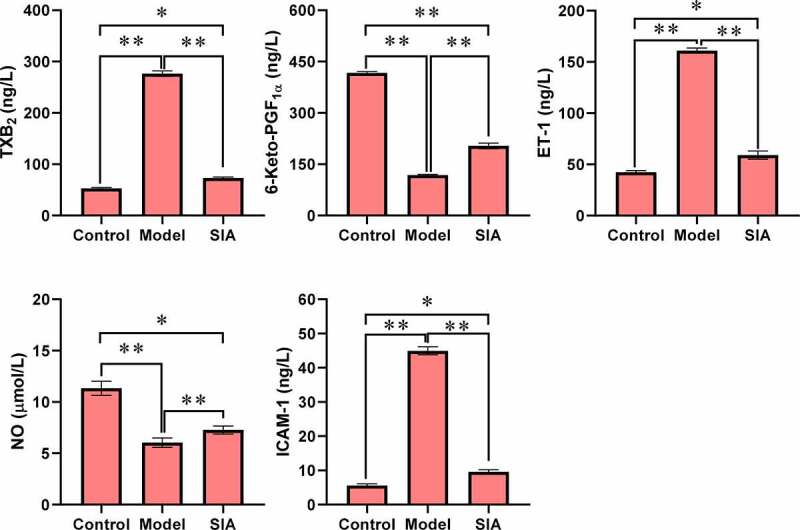
The levels of other blood vessel indicators. (*P < 0.05; **P < 0.01)

Figure 5 shows the levels of TXB2, 6-Keto-PGF1α, ET-1, NO, and ICAM-1. Compared with the control group, the TXB2, ET-1, and ICAM-1 levels of the model group and the SIA group increased, while the levels of 6-Keto-PGF1α and NO decreased, and the difference was notable (*P < *0.05). Compared with the model group, the levels of TXB2, ET-1, and ICAM-1 in the SIA group decreased, while the levels of 6-Keto-PGF1α and NO increased, and the difference was notable (*P < *0.05).

### 3.5 Serum VE-cadherin and sICAM-1 levels

**Figure 6.**
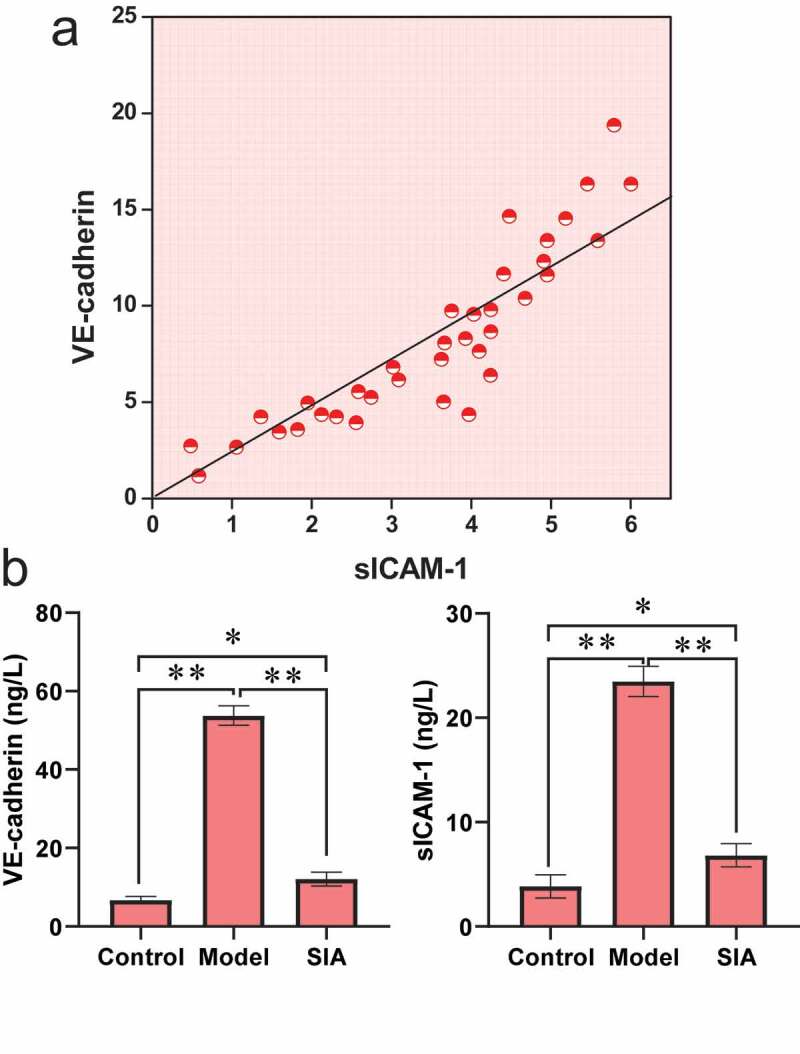
The serum VE-cadherin and sICAM-1 levels

Note: (a) correlation between VE-cadherin and sICAM-1; (b) VE-cadherin and sICAM-1 levels; *P < 0.05; **P < 0.01

Figure 6 shows the levels of VE-cadherin and sICAM-1. It was noted from Figure 6(a) that the VE-cadherin level was positively correlated with the sICAM-1 level (r = 0.631, *P < 0.01)*. It was noted from Figure 6(b) that compared with the control group, the VE-cadherin and sICAM-1 levels of the model group and the SIA group increased, and the difference was notable (*P < *0.05). Compared with the model group, the levels of VE-cadherin and sICAM-1 in the SIA group decreased, and the difference was notable (*P < *0.05).

## 4. Discussion

Interventional radiology refers to the diagnosis and treatment of diseases using medical materials such as guide wires, catheters, and embolic agents, under the guidance of X-ray, CT, and ultrasound images [11]. The stenosis or occlusion of the lower extremity arteries can cause ischemia, hypoxia, and cell necrosis of the distal arterial tissue [12]. The temperature-sensitive hydrogel is a liquid sol at a low temperature, and when the temperature exceeds its minimum dissolution temperature, it transforms into a solid gel [13]. Because temperature-sensitive hydrogel is characterized by non-toxicity and good biocompatibility, it has been widely used in the fields of biomedicine and pharmaceuticals. Additionally, it can also be used for gene carriers, controlled drug release and biological sensors [14,15]. Compared with linear polymer solutions, nanogel dispersions are characterized by low sol viscosity, high strength, and strong shear thinning ability [16]. In the study, the PIB nanogel, a temperature-sensitive liquid embolic agent, was prepared and its toxicity on cells was detected. The results showed that, the cell proliferation activity was always higher than 90% under different concentrations of PIB temperature-sensitive nanogel extracts, and the toxicity was Class I, meeting the national in vitro toxicity standards for biomedical materials [17]. Subsequently, the Calcein-AM/PI fluorescent staining was used to detect the toxicity on Hela cells directly exposed to PIB temperature-sensitive nanogels. Calcein-AM is highly lipophilic, and it can pass through the cell membrane, and stain the cell with green fluorescence [18]. PI solution cannot pass through the cell membrane of living cells, but it can penetrate the nucleus of dead cells and eventually is embedded on the chromosomes of the cells, staining the cells red [19]. The results of this study found that, the survival rate was always higher than 90% after direct contact with different concentrations of PIB temperature-sensitive nanogels. Taken together, the PIB temperature-sensitive gel in this study has good cell compatibility.

Next, an animal model of lower extremity arterial occlusion was prepared by injecting PIB temperature-sensitive gel into the high-fat feeding rabbit. The results found that the serum levels of TC, TG, and LDL in the model rabbit increased, while the HDL level decreased, indicating that the model was successfully constructed, laying the foundation for subsequent research. The animal model was treated by SIA. The results showed that, the serum levels of TC, TG, and LDL decreased after treatment, while the HDL level increased, but compared with healthy rabbits, there were notable differences in the above indicators. Studies have shown that, TXB_2_ can affect vasoconstriction and platelet aggregation, and when a thrombus exists, the body’s TXB_2_ and 6-keto-PGF1α levels increase [20,21]. This is in line with the results of this study in terms of the levels of TXB_2_ and 6-keto-PGF1α. ET-1 is a vasoconstrictor factor, and the biological effect of NO is opposite to that of ET-1 [22,23]. The increased expression of the adhesion molecule ICAM-1 can enhance the adhesion and transfer ability of macrophages, which is also a prerequisite for atherosclerosis [24]. The study has found that SIA can reduce the level of ET-1 and ICAM-, and increase the level of NO.

VE-cadherin is able to mediate the adhesion between cells of the same type, and it is the main adhesion protein of endothelial cells [25]. Studies have shown that VE-cadherin can maintain the stability of the internal environment and is instrumental in maintaining the integrity of normal vascular endothelium [26]. sICAM-1 is a soluble intercellular adhesion molecule, which can reflect the local expression of ICAM-1, and participates in the activation of endothelial cells and signal transduction [27]. The results of this study showed that the levels of VE-cadherin and sICAM-1 were increased, and the levels of VE-cadherin and sICAM-1 were decreased after SIA.

## 5. Conclusion

PIB thermosensitive nano-gel is an ideal agent for vascular embolization. SIA has a good effect on the treatment of lower extremity arterial occlusion. This article provides a reference for clinical treatment of lower extremity arterial occlusion. However, some limitations in the study should be noted. The sample size is small, which will reduce the power of the study. In the follow-up, an expanded sample size is necessary to strengthen the findings of the study. In addition, there is no comparison of indicators before and after treatment. Some intuitive images and data such as DSA images and more characterization analysis are required to obtain more detailed results.
